# A Review and Evaluation of Control Architectures for Modular Legged and Climbing Robots

**DOI:** 10.3390/biomimetics9060319

**Published:** 2024-05-27

**Authors:** Carlos Prados, Miguel Hernando, Ernesto Gambao, Alberto Brunete

**Affiliations:** Centre for Automation and Robotics (CAR UPM-CSIC), Universidad Politécnica de Madrid, 28006 Madrid, Spain; miguel.hernando@upm.es (M.H.); ernesto.gambao@upm.es (E.G.); alberto.brunete@upm.es (A.B.)

**Keywords:** control architecture, legged robots, modular robots, control structure

## Abstract

Robotic control is a fundamental part of autonomous robots. Modular legged and climbing robots are complex machines made up of a variety of subsystems, ranging from a single robot with simple legs to a complex system composed of multiple legs (or modules) with computing power and sensitivity. Their complexity, which is increased by the fact of needing elements for climbing, makes a correct structure crucial to achieve a complete, robust, and versatile system during its operation. Control architectures for legged robots are distinguished from other software architectures because of the special needs of these systems. In this paper, we present an original classification of modular legged and climbing robots, a comprehensive review of the most important control architectures in robotics, focusing on the control of modular legged and climbing robots, and a comparison of their features. The control architecture comparison aims to provide the analytical tools necessary to make informed decisions tailored to the specific needs of your robotic applications. This article includes a review and classification of modular legged and climbing robots, breaking down each category separately.

## 1. Introduction

In this review, we explore four key dimensions of robotics: control architectures, legged robots, climbing robots, and modular robots. By delineating these sections, we aim to provide a clear and coherent overview of each area, ensuring a smooth transition and a comprehensive understanding of these interconnected aspects. Our classification of control architectures is meticulously designed to highlight key attributes, such as modularity, robustness, implementability, versatility, and explicitness, each of which plays a critical role in the operational success of modular legged and climbing robots. By delineating these architectures based on such features, we intend to equip readers with the analytical tools needed to make informed decisions tailored to their specific robotic application needs.

An essential aspect of autonomous robots is their control architecture, which outlines the structural guidelines for a robot’s behavior. They determine the actions and movements that the robot needs to effectively perform to accomplish one or more objectives. Their primary aim is to organize the system effectively to ensure defined roles, modularization of system components, and high fault tolerance, striving to maintain control in a wide range of scenarios. Key features of robot control architectures are the ability to face multiple objectives at once, perform efficient sensor fusion, exhibit resilience to component malfunctions, adapt to new surroundings, easily expand and adjust its programming, autonomously make decisions based on its current state, and interact with the environment properly.

Control architectures are usually organized at different levels, maintaining a hierarchical structure, where higher levels make decisions that lower levels must execute. Levels are commonly composed of agents, understanding an agent as a computer system capable of autonomously acting in its environment to achieve its delegated objectives [1,2]. With respect to the control distribution, it is possible to differentiate between centralized and decentralized controls. Centralized control is defined by the synchronized behavior of agents, where their decisions are directly influenced by the status of other agents. In contrast, decentralized control allows agents to make decisions independently, without directly taking into account the condition of other agents in the system.

A control architecture is defined as a multi-agent system where the communication rules and protocols are well-defined. Robot control architectures are distinguished from other software architectures due to the special needs of robot systems. They operate in complex dynamic real-time environments and they have to control diverse sensors and actuators in real time, be robust under uncertainty and noise, monitor and react to unexpected situations, and do all this concurrently and asynchronously. Moreover, robot systems need to respond at varying temporal scopes from millisecond feedback control to minutes or hours for complex tasks.

Moving forward, we delve into the world of legged and climbing robots, examining their unique design principles and capabilities. Legged and climbing robots excel at navigating complex terrains with discontinuous surfaces, offering a higher resilience during stable motion due to their exceptional ability to maintain balance and quickly adapt [3]. Their design incorporates redundancy and rapid adaptation mechanisms, enabling them to operate in extreme conditions and areas inaccessible to humans or traditional locomotion methods. Although legged robots may consume more energy than their wheeled counterparts, their agility and adaptability make them indispensable for traversing unstructured landscapes [4]. In addition, their ability to climb equips them to carry out inspections in challenging locations, including wind turbines, skyscrapers, aircraft bodies, nuclear facilities, tunnels, and cooling towers [5]. In contrast, modular robots boast unparalleled versatility and configurability. Their modularity enhances the resilience of the system to failures, allows for easier scalability, and reduces production costs [6].

As we move towards an increasingly automated future, modular legged and climbing robots are poised to play a pivotal role in various sectors. Their unique combination of adaptability, resilience, and versatility enables them to tackle a broad spectrum of challenging or impossible tasks for humans or conventional robots. From performing precise and hazardous maintenance in industrial settings to conducting search and rescue operations in disaster-stricken areas, these robots can navigate and manipulate their environment with unprecedented efficiency and safety. In addition, their modular design allows for rapid customization and repair, significantly reducing downtime and operational costs.

This article is organized as follows: we first classify climbing, legged, and modular robots in Section 2. In this section, we introduce the concept of legged robots and classify some of them in Section 2.1. In Section 2.2 and Section 2.3, we propose a taxonomy for climbing and modular robots, respectively. We review some important control architectures applicable to modular legged and climbing robots chronologically in Section 3. In Section 4, we summarize and compare the control architectures described before with respect to some relevant features for modular legged and climbing robots. Lastly, we present our conclusions and final considerations in Section 5.

## 2. Classification of Modular Legged and Climbing Robots

The main challenges in modular, legged, and climbing robot development lie in the realms of physical design, energy efficiency, and robustness in diverse environments. Modular robots must achieve a balance between versatility and complexity, ensuring they can adapt to various tasks without becoming too complex. Legged robots face hurdles in mimicking the nuanced movements of their biological counterparts and require sophisticated mechanics to navigate in many terrains effectively. Climbing robots, meanwhile, must overcome obstacles related to adhesion, surface compatibility, and gravity. In the future, trends are expected to emphasize the integration of advanced materials and biomimicry to enhance adaptability and performance, along with the pursuit of miniaturization and energy optimization to extend operational capabilities and application domains.

**Figure 1:**
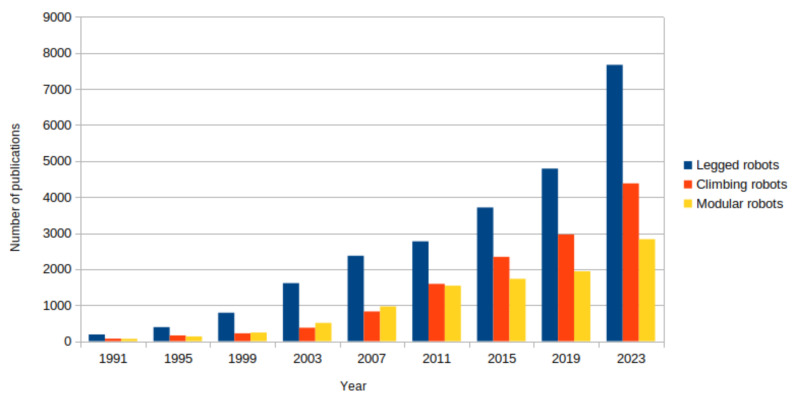
Triannual publication of bibliometric papers. Note(s): This figure represents the publication trend of bibliometric papers between 1991 and 2023. The data were retrieved from the Google Scholar database in the subject areas of “legged robots”, “climbing robots”, and “modular robots”.

The trend (triannual) in number of publications related to legged, climbing, and modular robots is shown in Figure 1. The number of publications has increased exponentially, emphasizing the greater volume of publications for legged robots compared to the rest.

In the following subsections, we delineate the categories of legged, climbing, and modular robots separately. This classification is designed to provide a structured and comprehensive analysis of each robot type within its context. This separation and focus are aimed at enhancing clarity and providing a detailed framework for understanding the distinct contributions and challenges associated with each type of robot.

### 2.1. Legged Robots

Legged robots differ from other mobile robots by using articulated limbs to provide locomotion. Compared to wheeled robots, they show more versatility by moving in many different terrains. However, they are more complex systems that consume more power. Despite their lower energy efficiency compared to wheeled robots, the flexibility and adaptability of legged robots make them invaluable for operating in environments lacking structure and predictability [4].

Legged robots can be classified into many categories according to different parameters. The most common ways to gather similar robots are:

- Depending on the application and capabilities: walking or climbing robots.
- According to the leg structure: articulated legs (with and without wheels), orthogonal legs, pantograph legs, or telescopic legs (Table 1).
- According to the number of legs: monopod, biped, quadruped, hexapod, etc.

The mechanical designs of legged robots draw significant inspiration from the structure of multilegged animals, utilizing principles of bionics in their research and development across all scales. For heavy-duty legged robots in particular, the demands on their mechanical structures are substantially higher [7]. The design of these structures critically influences their overall performance, affecting aspects such as mobility, energy efficiency, and the implementation of control algorithms. A recurring challenge in legged robotic mobility is the issue of low energy efficiency, often attributed to inadequate leg actuators [25]. This inefficiency is particularly pronounced in climbing robots due to the complexities in accurately estimating the necessary actuators’ torque. These calculations often fail to account for the hyperstatic complexities inherent in the design of such robots, exacerbating the problem.

**Figure 2:**
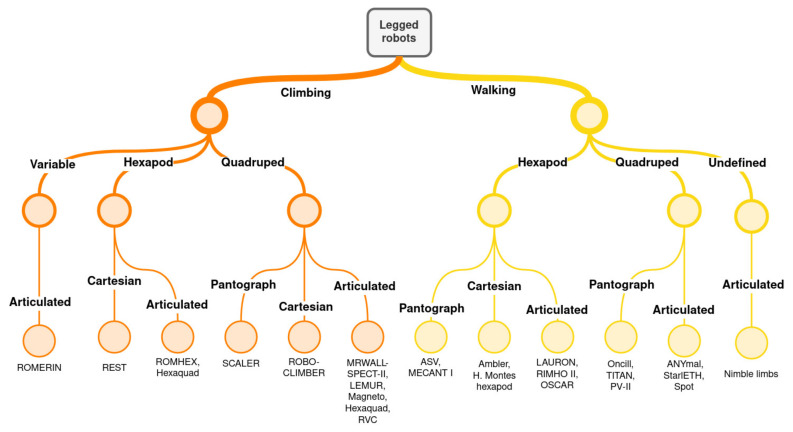
Legged robot classification. ROMERIN [5,26], REST [27], ROMHEX [28], Hexaquad [29], SCALER [18], ROBOCLIMBER [16], MRWALLSPECT-II [30], LEMUR [31], Magneto [32], RVC [33], ASV [34], MECANT I [22], Ambler [15], H. Montes hexapod [17], LAURON [8], RIMHO II [35], OSCAR [36], Oncilla [19], TITAN [37], PV-II [21], ANYmal [9], StarlETH [38], Spot [39], Nimble limbs [40].

Figure 2 shows a classification of some legged robots according to the parameters explained before. To reduce the size of the classification tree, only quadruped, hexapod, and variable-legged robots are included. It is worth mentioning that the use of legs for climbing involves the introduction of some variations in walking robots. Thus, simply attaching an adhesive system to the leg tips is insufficient for effective climbing robots. The kinematics and the arrangement of the legs must also be adapted consequently. This includes adjustments such as keeping the robot’s body closer to the surface it is climbing.

### 2.2. Climbing Robots

A climbing robot is a type of robot specifically designed to move across non-horizontal surfaces where typical wheeled systems would not be effective. Climbing robots have applications in various fields, including inspection and maintenance of structures (such as bridges, tall buildings, and wind turbines), surveillance, search and rescue operations in difficult terrain, and even in space exploration for maneuvering around spacecrafts or asteroids. Their design and functionality are tailored to the specific requirements of the task and the environment in which they are intended to operate, balancing factors such as weight, power consumption, adhesion method, and mobility to effectively perform their intended tasks.

Early evidence points to a significant improvement in productivity and safety in various dull, dirty, and/or dangerous services tasks [41] in which climbing robots are involved. These systems are adopted mainly when direct access by a human operator is very expensive due to a hazardous environment or the need for scaffolding. Usually due to their application, climbing robots carry instruments; therefore, they should have the capability to bear high payloads with lower self-weight [42]. The cleaning of the façades of high-rise buildings and skyscrapers, and inspection and maintenance tasks in large infrastructures and facilities, offer enormous opportunities for the use of climbing robots. In fact, in recent years a considerable number of climbing robots have been developed using various technologies and approaches [43,44]. These robots can be differentiated based on two principles: the adhesion system used and the type of locomotion on which they are based.

Regarding the **adhesion system,** many climbing robots employ either negative pressure vacuums or propeller mechanisms [45,46,47], utilize magnetic attachments when operating on metal surfaces [48,49,50,51], or depend on mechanical methods [52,53]. There are other adhesion technologies, such as elastomer [54,55] and electrostatic [56,57], but their use is more limited. Another type is the one used in the ROBOCLIMBER [16], which uses hydraulically operated traction devices to grasp and pull steel ropes.

Each adhesion method offers advantages and drawbacks, with suitability varying based on specific surface conditions and requirements [58]. Magnetic adhesion is energy-efficient and reliable but limited to ferromagnetic surfaces and complicates detection equipment usage. Elastomer and electrostatic methods work on various surfaces but are limited in reliability and carrying capacity. Vacuum adhesion is versatile and reliable for carrying detection equipment, provided the vacuum remains intact, although it is energy intensive and requires specific surface conditions.

Examples of **vacuum adhesion** are widely found in the literature. For example, in [59], the authors describe a robot that achieves swift vertical movement by employing a chain system with 24 suction pads on two tracked wheels, allowing for continuous and rapid climbing. Similarly, the work in [60] introduces a robot designed to ascend pillars and tubes by employing wheels that grip the interior surfaces of these structures, adapting to circular or near-circular cross-sections. In another study, [61] present a robot designed for inspecting industrial vessels using a pair of coaxial propellers mounted on a wheeled platform to navigate vertically while neutralizing drag forces. Furthermore, vacuum adhesion technology has been effectively implemented in various window cleaning robots, as evidenced by [62,63,64].

In fact, within architectural environments, pneumatic adhesion stands out for its adaptability. This category includes technologies such as passive suction cups [65,66], vacuum chambers exemplified by Alicia 3 [67] and the system introduced in [68], along with vacuum generation methods [69]. The latter technique is used in the ROMERIN robot, chosen for its compact design, flexibility, simplicity, adaptability, and effectiveness for different surface conditions.

The robot known as Magneto [32] serves as an illustration of a climbing robot utilizing **magnetic adhesion** technology. It shares certain kinematic and design characteristics with the ROMERIN robot, including a compliant foot mechanism with three degrees of freedom (DOF). Magneto is capable of navigating through openings as narrow as 23 cm. A further example of magnetic adhesion technology is detailed in [70], which describes the development of magnetic gripping devices for the HyReCRo robot. This bipedal robot is designed to climb and survey steel structures. This method is unique because each gripper is equipped with three switchable magnets, which can be attached or detached from a ferromagnetic base by altering their mutual orientation. The OmniClimbers robot [50] utilizes omnidirectional wheels [71] to inspect flat and convex human-made ferromagnetic structures with magnetic adhesion. In [72], a wall-climbing robot designed for large steel structures features a shape-adaptive magnetic adhesion mechanism, incorporating a rotational device in each wheel to adjust the magnets’ orientation, ensuring the magnetic force remains perpendicular to the surface. Additionally, ref. [73] introduces and validates a novel class of climbing robots that can adhere to vertical surfaces, including non-ferromagnetic and curved ones, using permanent magnets, expanding their climbing capabilities.

**Mechanical adhesion,** which relies on gripper-based mechanisms, is noted for its rather narrow range of application, primarily due to the specific environments it targets. This has led to its diminished utilization. Examples of robots employing this type of adhesion include ROMA I [74], SCALER [18], and LIBRA [75]. Additionally, there has been a noticeable increase in the adoption of electrostatic systems. These systems are inspired by geckos [76] and make use of microspines, which are an array of tiny spines that latch onto surface asperities [77] or leverage dry adhesives for attachment [78].

Concerning the **type of locomotion** (wheeled or legged robots), the main challenges are stable climbing ability and he ability to overcome obstacles. Wheeled robots, like those discussed in [79] and LARVA [80], offer speed and stability, but struggle with obstacle navigation. In contrast, legged robots, while slower and challenged by locomotive stability, excel at navigating obstacles and are adept at moving through complex structures, including those found in civil infrastructure. Legged and climbing robots with limited DOF face challenges in transitioning between planes. Enhancing the DOF count introduces complexity but simultaneously increases adaptability and climbing proficiency.

**Figure 3:**
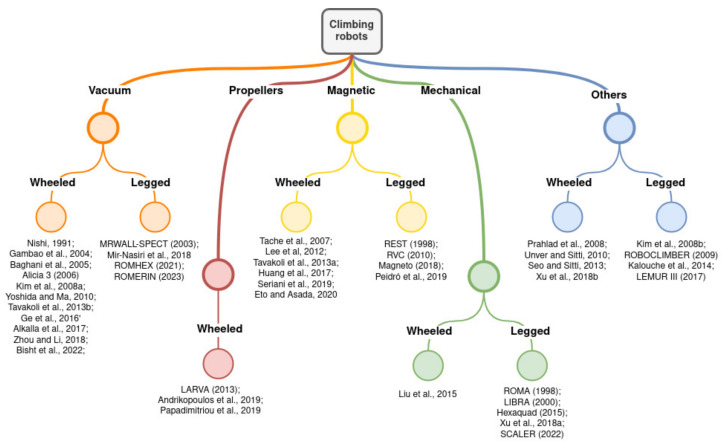
Climbing robot classification (citations included from top to bottom). Vacuum wheeled: [46,47,50,59,60,61,62,65,66,67]. Vacuum legged: [12,28,63,81]. Propeller wheeled: [69,79,80]. Magnetic wheeled: [48,49,50,51,72,73]. Magnetic legged: [27,32,33,70]. Mechanical wheeled: [53]. Mechanical legged: [18,29,52,74,75]. Others wheeled: [52,54,55,57]. Others legged: [16,59,77,82].

Figure 3 shows a classification of most of the climbing robots mentioned in this article according to the parameters explained above.

### 2.3. Modular Robots

Modular robots are designed with parts that can be reconfigured to assume different shapes and functions. A module can be defined as a component that is repeated in a construction of any kind to make it easier, more regular, and more economical. Thus, a robotic module is a “module that performs, totally or partially, typical tasks of a robot and that can interact with other modules” [6]. Key benefits of employing modular robots include enhancing the system’s versatility and configurability, achievable through either manual adjustments or autonomous reconfiguration. Such systems also exhibit increased fault tolerance and scalability. In addition, they help reduce production expenses, as the need for mass production is limited to one or a few types of modules, thereby eliminating the need for assembly among different parts. One important characteristic in modular robots is the possibility of cooperation amongst modules to achieve certain tasks. For example, to perform different locomotion patterns, or in case of failure of one module, correct the error by completing the task.

Modular robots offer enhanced versatility due to their reconfigurable nature, allowing for quicker assembly, maintenance, and replacement. Nevertheless, their capacity to adapt to varied applications introduces an additional layer of complexity in control, stemming from the need for generalized solutions. As the quantity of modules escalates, the computational demands for numerous tasks increase significantly, leading to a rise in complexity [83].

The literature differentiates between those modular robots that maintain connections to programmable matter systems capable of performing arbitrary shapes on demand [83], called self-reconfiguring modular robots, and those whose modules serve as a component whose location is constant during operation, called modular manually reconfiguring robots. Modular self-reconfiguring robots represent a class of autonomous kinematic machines characterized by their ability to alter their form. Unlike traditional robots that have a fixed shape and are equipped with standard actuation, sensing, and control systems, these robots possess the additional capability to intentionally modify their structure. They achieve this by changing how their components are connected, allowing them to adapt to different situations, assume new functions, or repair themselves after suffering damage [84].

For both self-reconfiguration and manual reconfiguration, modules must maintain a high degree of autonomy in mechanical, electrical, powering, and control aspects [85]. Achievements have been made in terms of mechanical cooperation for movement, electrical interaction for module-to-module communication, and control for distributed computing and decision-making. However, when it comes to power-sharing between modules, significant challenges remain. Currently, most modular robots are energized either through external tethers or their own battery systems, with lithium–polymer and lithium–ion batteries being the most common sources [6]. The concept of power sharing initially emerged in the context of Atron modules [86], where it was used more as a means of distribution of power rather than sharing. The notion of “energy homeostasis” was introduced in the Symbricator Project [87], describing a mechanism for managing the flow of energy among the system’s modules. This process aims to ensure the robotic organism can sustain itself for extended periods without external intervention, by efficiently distributing power within the system. Other relevant modular robots with power-sharing capabilities are the Superbot modules [88], or the recent [89,90].

Among **self-reconfiguring** modular robots, we can highlight Polybot [91], Crystalline [92], M-TRAN [93], Telecube [94], Atron [86], Superbot [88], Molecubes [95], Roombots [96], Ubot [97], Transmote [98], CoSMO [99], Hinged-Tetro [100], SB blocks [101], Nimble Limbs (NL) [40], Morphius [102], MLS [103], or KARAKASA [104]. On the other hand, among **manually reconfiguring** modular robots, we consider relevant Conro [105], Microtub [106], Odin [107], iMOBOT [108], $M^{3}$ Express [109], Kairo 3 [110], Fable II [111], TR: R [112], Snapbot [113], and WalkingBot [114].

All robots mentioned are included in Table 2, which analyzes if modules are mechanically homogeneous, if they share data and power, and if they are decentralized controlled.

**Figure 4:**
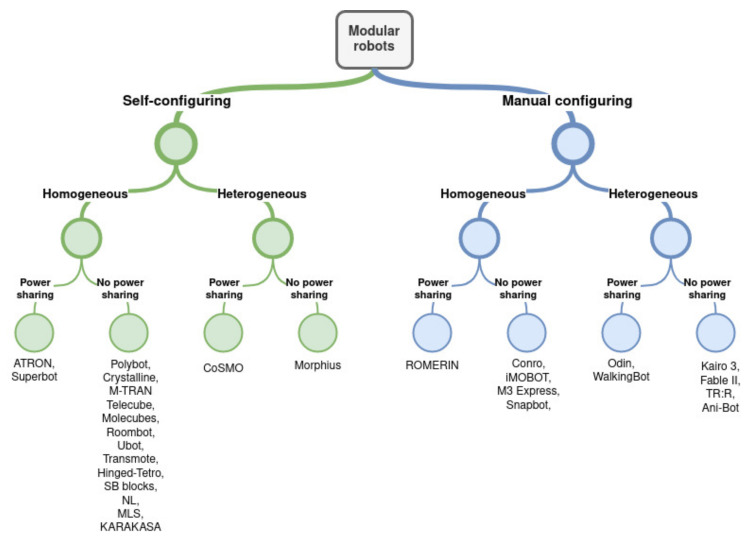
Modular robot classification.

Figure 4 shows a classification of some modular robots that have been mentioned in this article according to the parameters explained above. To reduce the size of the classification tree, only mechanical homogeneity, power-sharing capabilities, and self-configuring features are included.

Roombots serve as a notable example of modular robotics, featuring modules with rotational DOF for movement and active connectors for dynamic reconfiguration during operation [96]. These modules coordinate through neural networks using central pattern generators (CPG), which create synchronized rhythmic patterns autonomously, without relying on rhythmic inputs from sensory feedback or higher-level commands [115].

To encapsulate, modular robots are designed to alter their structure to suit various tasks and environments [83,116]. These systems consist of uniform components that collaborate to fulfill a shared objective. When multiple modules are integrated into a larger system to undertake more complex functions, this ensemble is often referred to as a robotic organism. This concept parallels living systems, where the organism operates as a unified entity. The aim behind using a collective of simpler robots, or a robotic organism, is to enhance the overall robustness and adaptability of the system [87].

## 3. Robot Control Architectures

Shifting from discussing recent progress in modular, legged, and climbing robots to exploring control architectures, we find the central theme that unites this article. The initial section not only showcases the recent developments in the physical design and capabilities of these robots but also prepares the ground for a deeper exploration into their control architectures that define their behavior and performance. This progression from the evaluation of mechanical attributes to the analysis of control mechanisms is interconnected to highlight how their control systems largely determine the functionality and efficiency of these robots. The comparative analysis of various control architectures is, thus, not merely a catalog of options but a critical discourse on how these frameworks can amplify or constrain the robots’ operational efficacy. Therefore, the importance of not only a good mechanical design for a specific application is presented, but also a good design of the control system seeking, as far as possible, characteristics such as modularity, versatility, or robustness.

**Figure 5:**
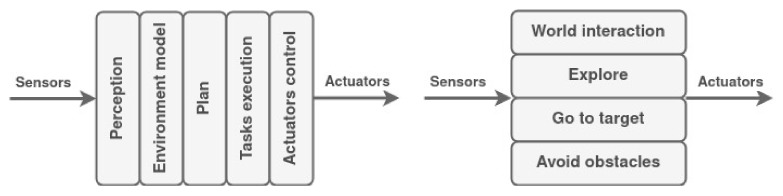
Structure of deliberative (**left**) and reactive (**right**) control architectures.

Robot architecture and programming began in the late 1960s with the Shakey robot at Stanford University [117]. It was divided into three functional elements related to the three basic elements of control, (1) perception, which models the environment based on the sensory data, (2) planning, which plans the task steps, manages the resources, and monitors the activity, and (3) the action, which performs the navigation, the detailed pacification of the movements and actions, and controls the actuators. Depending on the organization of these basic elements, a control architecture may be classified as deliberative, reactive (Figure 5), or hybrid.

Deliberative control architecture generates an optimal plan given a specific goal; however, this way is distant for robots working in situations with foreseen aspects, rough terrains, or unknown hazards. These architectures produce slow responses with high computational costs, so it is proper when the world is static and the task is well-defined. On the other hand, reactive control architectures appear to be a good alternative when the system is expected to have relative dependability. This architecture produces fast responses with low computational cost. However, there is a strong dependency on the sensory data, and the best solution is not guaranteed.

Since this first version of the control architecture, there have been many approaches to building a functional system for a given machine. In this section, we explore some important control architectures for modular legged and climbing robots that have led the way for the control of this type of robot.

### 3.1. SFX-EH

The Sensor Fusion Effects Exception Handling (SFX-EX), first presented in [118] and improved in [119], implements an exception handling strategy divided into two steps:

1. Error classification, which generates hypotheses about the underlying cause of the failure. It follows the following procedure: (a) generate all possible causes based on the symptom. (b) Order the list of associated tests and execute them to confirm any of these causes. (c) Terminate classification when all tests have been performed or an environmental change has been confirmed.
2. Error recovery, which attempts to replace the logical sensor with an alternative. If there is no other option, the mission is deemed unsuccessful, and the robot planner is given control.

**Figure 6:**
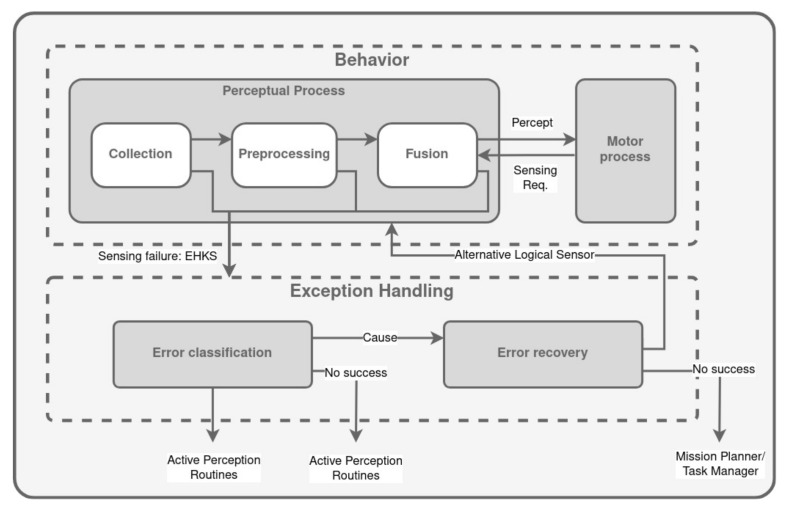
Overview of SFX-EH. Adapted from [119].

Figure 6 shows a conceptual layout of the sensing activities in SFX-EH. The perceptual process component of a behavior is executed in three steps. First, observations are collected from each description on the logical sensor. The descriptions are then pre-processed to compensate for asynchronous observations. The fusion step integrates the evidence for the perception from each description and passes it to the motor process.

The core mechanism for identifying sensing failures relies on internal self-monitoring processes within a behavior, while the option for monitoring at higher behavioral and planning levels is also considered. SFX plays a key role in this process by regularly evaluating the data for potential problems after each step. SFX identifies four distinct symptoms of sensing failures: missing data (the description has not been updated with a new reading), highly uncertain data (the observation of a description is vague or ambiguous), highly conflicting observations (the observations from multiple descriptions do not show a consensus), and below minimum certainty in the perception (the evidence that the perception is correct is too low for the motor process to safely use). If a definitive error is detected, the behavior’s perceptual processing is immediately paused, triggering the activation of any recovery procedures if available, or transferring control to the exception handler for appropriate action.

The module for managing exceptions operates at a global level. It relies on the Exception Handling Knowledge Structure (EHKS) to provide pertinent information about the sensing failure and the task at hand. The EHKS takes the form of a frame that contains six distinct slots. The failure step slot serves as a flag that indicates whether the failure occurred during a particular stage of execution. The error slot outlines the specific failure condition encountered. Within the body of evidence slot, an array of frames is stored, with each frame containing data from individual descriptions in the logical sensor. The environmental preconditions slot also holds an array of frames, each of which characterizes an environmental attribute that acts as a prerequisite to utilize a given sensor. This frame includes the anticipated value of the environmental attribute for the sensor’s optimal performance and references to other sensors sharing the same environmental requirement.

### 3.2. CIRCA

In the Cooperative Intelligent Real-Time Control Architecture (CIRCA), the authors integrate an artificial intelligence subsystem to reason about task-level problems that require its powerful but unpredictable reasoning methods. At the same time, a separate real-time subsystem uses its predictable performance characteristics to deal with control-level problems that require guaranteed response times. The key difficulty with this approach is to allow the subsystems to interact without compromising their respective performance goals [120].

**Figure 7:**
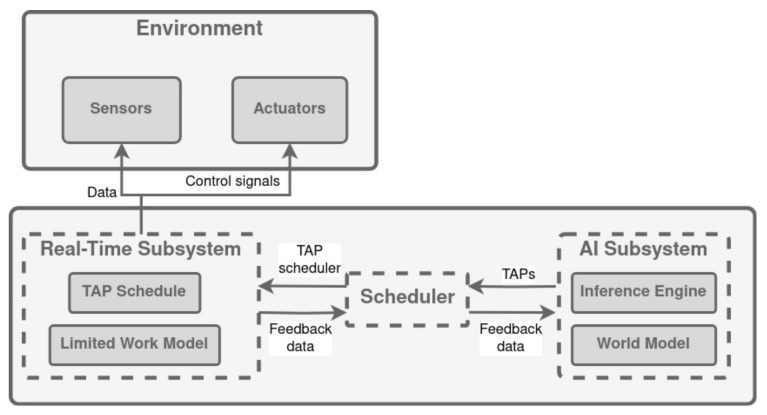
CIRCA architecture. Adapted from [120].

The architecture scheme is shown in Figure 7. The Real-Time Subsystem (RTS) is responsible for implementing the actual guaranteed responses. The Artificial Intelligence Subsystem (AIS) and the Scheduler collaborate to fine-tune the subset of responses that the RTS supports. Both work towards ensuring that the entire system not only adheres to strict deadlines but also comes as close as possible to achieving system objectives. The RTS follows a cyclic schedule of uncomplicated test–action pairs (referred to as TAPS), each with established worst-case execution durations. Given that the RTS is exclusively dedicated to this purpose, it can ensure that the scheduled tests and actions are completed within predictable time limits.

The AIS engages in reasoning about the RTS’s confined reactivity, aiming to identify a subset of TAPs that can be reliably committed to meeting control-level objectives and progressing towards task-level goals. In tandem, the Scheduler contemplates the finite computational resources at the RTS’s disposal and constructs the TAPS’ schedule. As the AIS and RTS operate asynchronously, the AIS is not constrained by the stringent performance constraints that the RTS employs to ensure compliance with hard deadlines. Consequently, the AIS can employ uncertain heuristics with substantial variability without jeopardizing the system’s capacity to satisfy real-time deadlines.

### 3.3. ORCCAD

**Figure 8:**
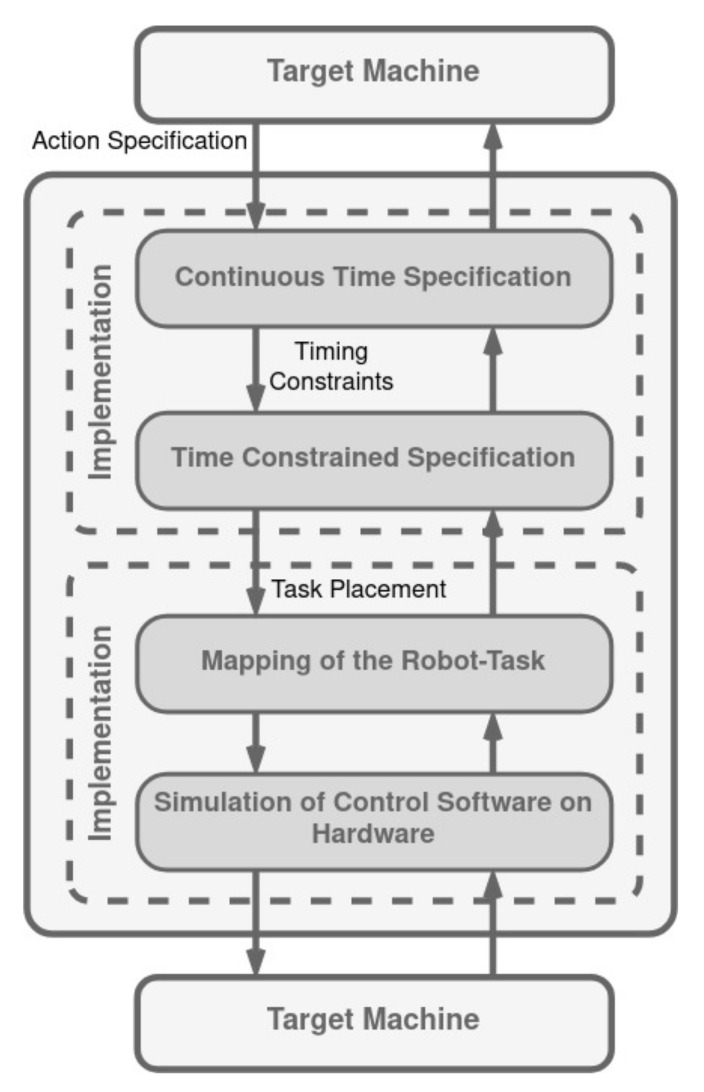
ORCCAD architecture. Adapted from [121].

Open Robot Controller Computer Aided Design (ORCCAD) is a development environment for specification, validation by formal methods and by simulation, and implementation of robotic applications (Figure 8). This architecture gives much freedom to the control designer in order to match an end-user specification. Thus, the objective of the ORCCAD system is to help the user to exploit this freedom in the most efficient way. The inherent nature of the corresponding controller is inherently open, as proficient users hold privileges to access every tier of control: the application layer is made accessible to the end user of the system, the control layer is configured by an automatic control specialist, and the lowest one, the system layer, is supervised by a system engineer [121]. Although the authors present the architecture as a three-level architecture, often the defined application layer is considered out of the architecture, and ORCCAD is contemplated as a two-layer architecture.

According to [122,123], an application is a set of actions that the system performs to reach a specified goal that an end-user has set. In this way, the specifications of a robotic application have to be modular, structured, and accessible to users with different expertise. Two different entities are defined in ORCCAD:

1. The Robot-task (RT). It represents an elementary robotic action, where automatic control aspects are predominant, although coherently merged with behavioral ones.
2. The Robot-procedure (RP), which is a basic element where only behavioral aspects are considered.

A robotic application is considered fully specified if all RTs needed by the application are identified, specified, and organized hierarchically in the form of RPs [124].

The RT represents the finest level of detail that an end-user interacts with at the application layer, and simultaneously, it is the broadest level of detail that a control systems engineer deals with at the control layer. The characteristics of an RT are fully defined by its temporal attributes, which are established by structuring each RT around specific real-time computing processes known as Module-tasks (MTs). These tasks, which are mostly periodic, execute the computations necessary for the control algorithm. Additionally, there are observer tasks dedicated to monitoring specific conditions and managing preconditions, exceptions, and post-conditions. The collection of MTs, along with their temporal attributes and the selected method of synchronization, constitute the Time-Constrained Specification (TCS) of an RT [121]. The RT’s non-periodic, reactive behaviors are managed by a distinct MT named the Robot-task Automaton (RTA). This special task is activated by signals originating from the RT, specifically through the observers’ outputs, and it facilitates the connection between the RT and the application-level input/output signals [124].

### 3.4. LAAS Architecture

**Figure 9:**
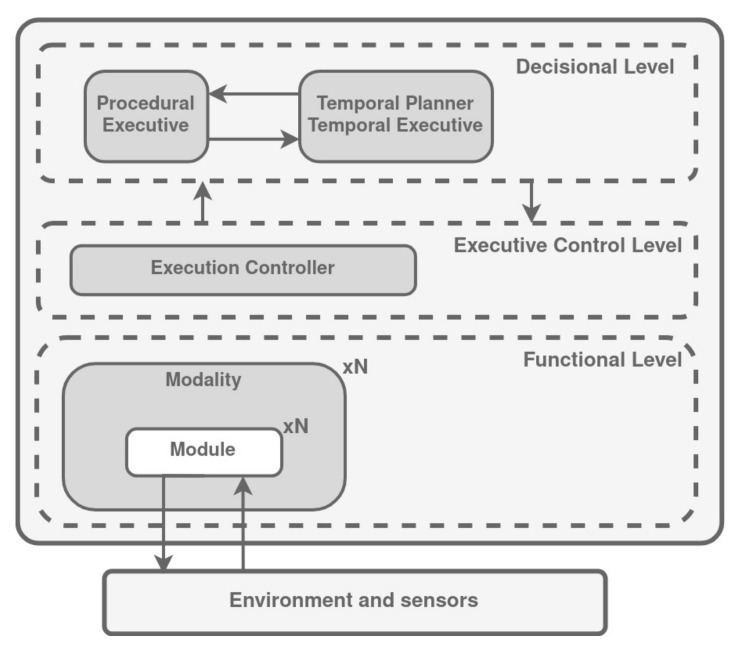
The LAAS architecture. Adapted from [126].

The Laboratory for Analysis and Architecture of Systems (LAAS) is an architecture for reflexive autonomous vehicle control [125]. This architecture enables the integration of processes with different temporal properties and different representations. LAAS architecture, shown in Figure 9, decomposes the robot software into three main levels (functional, decisional, and execution control level), having different temporal constraints and manipulating different data representations.

- Functional level: it includes all basic built-in robot action and perception capabilities. These processing functions and control loops are encapsulated into controllable communication modules. Each module provides services which can be activated by the decisional level according to the current tasks, and exports posters containing data produced by the module and for others to use.
- Decisional level: this level includes the capacities of producing the task plan and supervising its execution, while being at the same time reactive to events from the functional level.
- Execution control level: it functions like the interface between the decisional and the functional levels. It controls the proper execution of the services according to safety constraints and rules and prevents functional modules from unforeseen interactions leading to catastrophic outcomes.

### 3.5. CLARAty

The Coupled Layer Architecture for Robotic Autonomy (CLARAty) was developed to enhance the modularity of system software, simultaneously ensuring a tighter integration of autonomy controls. As outlined by the creators of CLARAty, conventional architectures for robots and autonomous systems are structured around three tiers: the Functional layer, the Executive layer, and the Planner layer.

**Figure 10:**
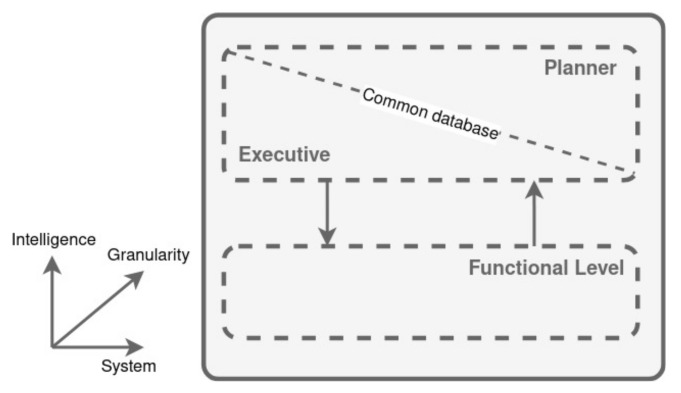
CLARAty architecture. Adapted from [127].

To address the shortcomings identified in the traditional three-level architectural framework, the proponents of CLARAty suggest a two-tiered architecture, as depicted in (Figure 10). The advantages of this refined architecture over the conventional three-tier model, as detailed in [127], include the introduction of an explicit third dimension that represents the granularity of the system layers. This approach aims to decrease granular sizes, effectively dividing the architecture into a greater number of smaller blocks, and facilitates the integration of both declarative and procedural approaches in the decision-making process.

The introduction of a granularity dimension in the CLARAty architecture allows for a clear depiction of the system’s hierarchical organization within the Functional Layer. Using an object-oriented hierarchy, the architecture visually demonstrates how subsystems are nested within one another, ensuring that fundamental capabilities are accessible at every level of this nesting [127]. In the context of the Decision Layer, this notion of granularity is reflected in the creation and execution of activity timelines. Given the intrinsic link between the physical system managed by the Functional Layer and the Decision Layer’s timeline granularity, there exists a significant correlation between the granularity of the system architecture and the granularity of the decision-making timelines.

The Functional Layer is both an interface between the software and the hardware, and an interface for the Decision Layer to access the basic capabilities of the system.

The Decision Layer breaks down high-level goals into smaller objectives, arranges them in time due to known constraints and system state, and accesses the appropriate capabilities of the Functional Layer to achieve them.

The following options are available depending on the ways in which the two layers are connected:

- A system with a very capable Decision Layer and a Functional Layer that provides only basic services.
- A system with a very limited Decision Layer that relies on a very capable Functional Layer to execute robustly given high-level commands.

The interaction of the two architectural layers can also be understood by considering the creation and execution of activities on a time-line.

### 3.6. RA

**Figure 11:**
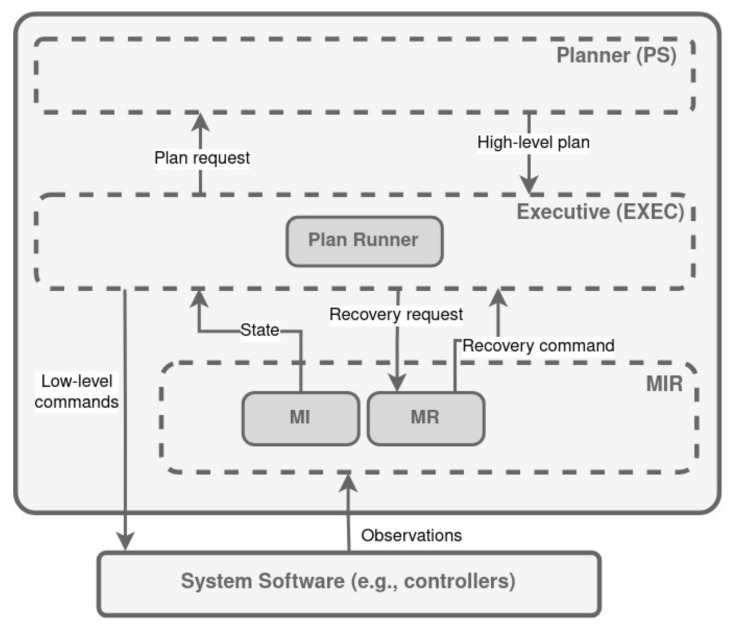
Remote Agent’s layered architecture. Adapted from [128].

The Remote Agent (RA) is an autonomous control system capable of closed-loop commanding of spacecraft and other complex systems [128]. It comes from the New Millennium Remote Agent (NMRA) architecture, first proposed in [129] and further extended in [130]. Within three layers, NMRA integrates traditional real-time monitoring and control with (a) constraint-based planning and scheduling; (b) robust multi-thread execution; and (c) model-based diagnosis. Similarly, the RA architecture integrates three layers of functionality, as shown in Figure 11: a constraint-based planner/scheduler (PS), a reactive executive (EXEC), and a Model Identification and Recovery system (MIR).

The planner (PS) is found in the higher level. It defines the state machines and the temporal constraints essential for generating feasible plans. Positioned beneath the PS, the Executive (EXEC) serves the critical function of converting the plan’s high-level directives into a sequence of timed, low-level instructions directed towards the System Software. This translation process begins with the EXEC’s plan runner module, which meticulously interprets the plan one action at a time. For every action during execution, the plan runner evaluates if all the specified logical and temporal conditions for concluding the action have been met. Upon confirmation, the action is terminated in full, its completion is communicated across the plan, and the initiation of the subsequent action is triggered. During the execution phase of an action, the EXEC model is used to run a specific procedure associated with that action [128].

EXEC depends on the MIR framework for essential support in interpreting low-level sensor data and processing commands. Within MIR, two principal functionalities emerge: Mode Identification (MI) and Mode Recovery (MR).

The role of MI is to accurately assess the current state of the system and inform EXEC of any state transitions. This process involves a comprehensive model of the system’s components, often requiring the consideration of interactions across multiple subsystems to accurately determine the condition of a specific device.

On the other hand, MR leverages the same detailed model utilized by MI to identify the most efficient route from the currently estimated (and possibly faulty) system state to the desired state, as dictated by EXEC to align with the overarching plan. In doing so, MR ensures that any steps taken toward recovery steer clear of invalid states, as indicated by EXEC, thereby safeguarding the integrity of the operational path of the system.

### 3.7. IDEA

**Figure 12:**
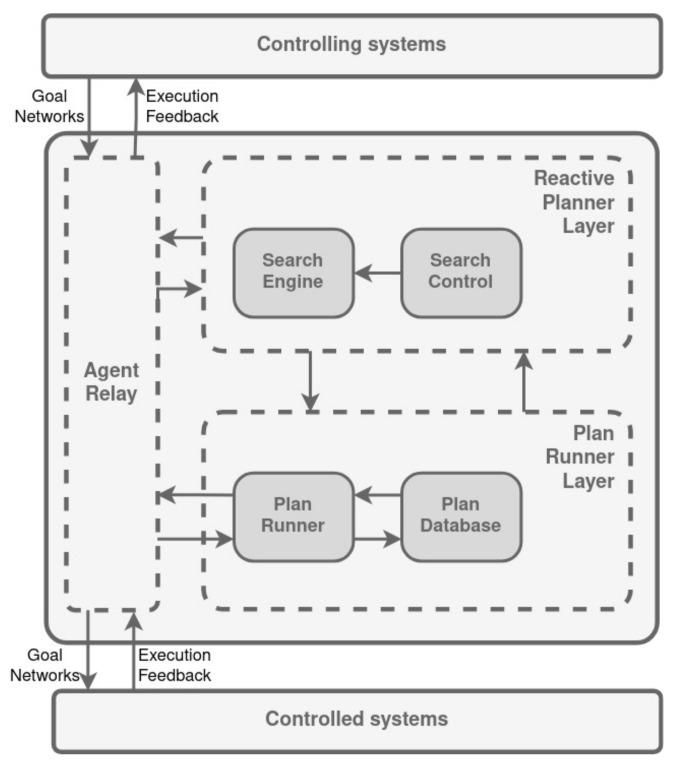
Structure of an IDEA agent. Adapted from [128].

The Intelligent Distributed Execution Architecture (IDEA) was created with the objective of creating a duplicate of RA architecture within a unified agent framework where all layers have the same structure. The scheme of this structure is shown in Figure 12.

The basic execution element within this context is referred to as a token which represents a specific time interval designated for the execution of a procedure by the agent [128]. A procedure (P) may have inputs (i), modes (m), and outputs (o) arguments and a status value (s). At any time during its execution, a procedure returns a value for each output.

The tokens are started sequentially. When a token is finished, a procedure returns a value for the status, and a new token is started. The process proceeds until it encounters one of two scenarios: (1) a status value is issued; or (2) the agent opts to halt the execution of the token. The moment this occurs is referred to as the token termination time.

Although inputs, outputs, and status play an active role in the execution of a token, the mode arguments are not monitored at execution. In contrast, they can be arbitrarily modified by a planning activity at any time during the agent’s problem solving.

The agent’s interaction with fellow agents is facilitated through a communication wrapper. This wrapper’s primary role is to transmit messages that trigger the execution of procedures by other agents or to receive objectives that the agent interprets as tokens. Parameters encompassing arguments, initiation times, and completion times for each received token are considered as inputs for the IDEA agent’s internal problem-solving process, guiding its subsequent actions. The IDEA agent boasts the capability to engage with numerous controlling and controlled agents, thus fostering multi-faceted communication networks [128].

The structure of permitted communications adheres to the directives established by the central Model, which outlines the viable exchange of procedures with external agents. This Model not only dictates the feasible procedures for interchange, but also outlines the prerequisites for sending goals to other agents (input arguments) and the anticipated feedback from agents executing tokens (output and status arguments).

Execution of tokens by the IDEA agent occurs exclusively after they have been incorporated into a plan preserved within a central Plan Database. This can be triggered either by goals forwarded from controlling agents or by internally generated subgoals.

The Plan Database maintains records of recent events, tokens currently in progress, and future tokens, encompassing various potential execution paths. Each token parameter corresponds to an associated variable, all interconnected by explicit constraints within a comprehensive constraint network.

At the heart of the agent’s operation lies the Plan Runner, an extension of the RA plan runner (as described in Section 3.6). This component operates asynchronously, activated by incoming messages or internal timers. Upon activation, the Plan Runner integrates received messages into the Plan Database and immediately engages a Reactive Planner. The role of the Reactive Planner is to produce a locally executable plan, ensuring both token parameter consistency with plan constraints and alignment with the domain model.

Validation of model support for a token involves confirming that a new token initiates when its immediate predecessor concludes along the timeline. Before starting a new token and invoking its procedure, the Reactive Planner verifies if the token aligns with the model as specified in the plan.

The overall cycle duration of the Plan Runner and Reactive Planner is restricted by a fixed timeframe, the execution latency. The Plan Runner is anticipated to awaken, process incoming messages, consult the Reactive Planner, receive notification of task completion, forward relevant messages to external agents, and then pause within the confines of the execution latency. Failure to adhere to this timeline results in agent malfunction and contingency measures will take effect.

IDEA accommodates diverse planning modules within a single agent, including Reactive and Deliberative Planning. Each module operates under a distinct internal logic and scope, yet adheres to the same input/output protocol: given an initial plan database, a planner constructs a fresh plan database conforming to specified quality criteria.

### 3.8. CMTI

**Figure 13:**
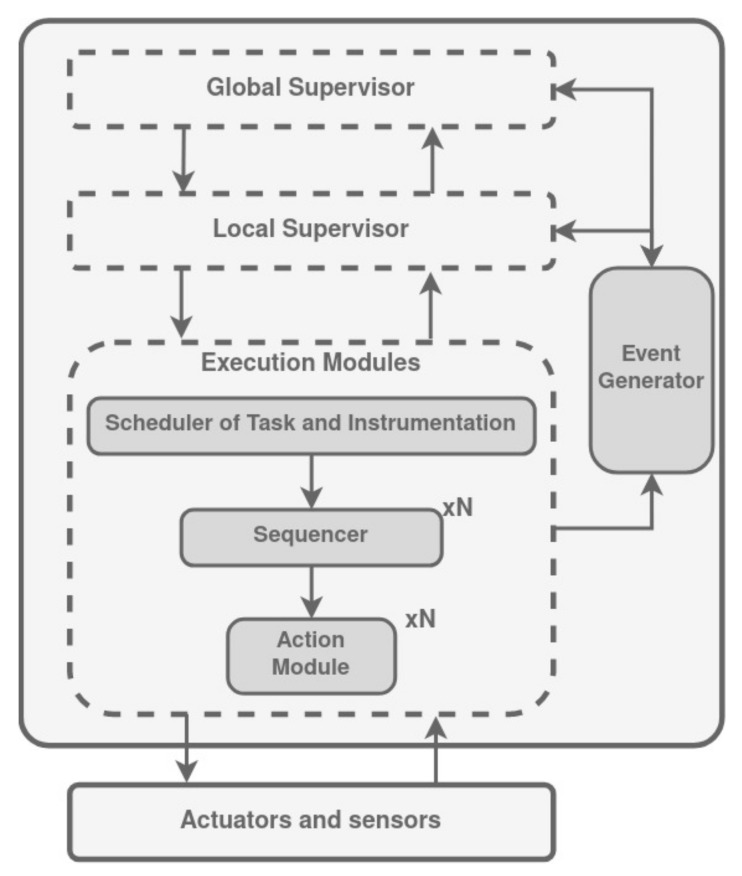
Control architecture of CMTI. Adapted from [131].

Contextual Management of Tasks and Instrumentation (CMTI) is a mixed architecture between deliberative and reactive architectures [131], originally intended for an autonomous underwater vehicle (AUV) and presented in [132]. As shown in Figure 13, it is organized into three layers: global supervisory control, local supervisory control, and low-level control. The higher level, the global supervisor (GS), is in charge of strategic mission management. Decisions related to robotic tasks to launch at a precise time, order of tasks, and mission plan changes are taken within this level. The second level contains local supervisors (LS). An LS checks resource availability, reacts to events that require fast response, and pilots low-level modules. The low level works cyclically. Several modules within this level are coordinated to configure sensors to compute controls, to manage instrumentation conflicts, etc.

Events that reflect a dangerous situation and need a fast response are built by the Event Generator (EG) from data sensors. EG is located between the LS and the low-level modules and monitors the sensor data to detect relevant events for LS or GS. Events that induce re-planning are sent to the GS and the other to the LS.

The communication between levels is done by exchanging data objects: objective, sub-objective, and order. The mission plan is described by the operator as a set of objectives that are then arranged to form a sequence. The GS receives from the user a file containing this set of objectives and, according to the state of the system, it selects objectives that must be launched. When an objective is selected to be executed, it is decomposed into a sequence of sub-objectives. A sub-objective achievement will require a set of control law and sensors. To perform a sub-objective the LS sends orders to the low-level modules. An order contains a list of tasks that will be repeated, at a given period, until the sub-objective achievement.

### 3.9. COTAMA

**Figure 14:**
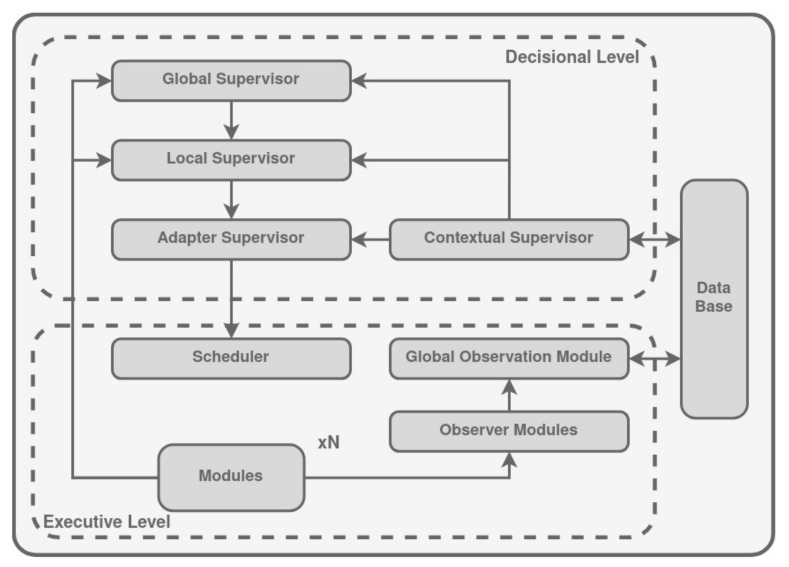
COTAMA architecture. Adapted from [134].

Contextual Task Management Architecture (COTAMA) (Figure 14) is a control software architecture that was released as an improvement to CMTI [133]. It has the objective of improving the reliability and robustness of the fault-tolerant mechanism for fault detection and fault recovery.

It is layered into two levels: Decisional level and Executive level. The Executive level is composed of a scheduler and low-level modules. There are three types of low-level modules mounted over a middleware which support the communication between them (e.g., ROS):

1. Control modules, which embed robotic algorithms (e.g., path planning or a location system).
2. Functional modules which implement specific functionalities (e.g., in/out ports).
3. The specific Observer modules that implement fault detection.

The COTAMA architecture consists of Observer modules generating observation data for the Global Observation Module (GOM), which diagnoses faults and identifies faulty modules based on this data [134]. The GOM’s diagnosis considers detected faults and current module status, estimating active functionalities and modules. The functional status of modules is represented by a status vector, updated with changes in context.

The Scheduler manages module activation and real-time constraints. It allocates execution time, ensuring time limits are not exceeded. If a module is frequently delayed, real-time faults are detected. The duration of sub-objective execution duration is also monitored, identifying real-time issues. Problems are reported to the decisional level.

COTAMA dynamically reconfigures the module parameters, interconnections, and scheduling for adaptation. Decision-making occurs at the Decisional level with Global and Local Supervisors. Contextual and Adapter Supervisors handle fault recovery. The Global Supervisor (GS) oversees mission execution and defines objectives for the Local Supervisor (LS) based on mission, environment, and robot state. The GS also ensures a safe state in fatal failures. The Local Supervisor manages objectives by dividing them into sub-objectives controlled by a scheduler. Sub-objectives consist of modules for specific tasks and are executed based on context and autonomy modes. The LS handles human–robot interactions for objective-level fault tolerance.

The Adapter Supervisor (AS) manages sub-objective modes and autonomy levels. It can adjust module parameters or switch to a degraded version of a sub-objective. The Contextual Supervisor (CS) focuses on fault recovery. It gauges robot context using the current state, mode, and functionalities, correlating with the current sub-objective. The CS selects suitable reactions on the basis of the module status from the Global Observation Module.

CS decisions depend on defined context severity, triggering dedicated events. Adapter events occur for mild or moderate failures, adapting sub-objective with modified low-level modules. Local events notify the LS of sub-objective infeasibility (hard failure). Global events alert the GS when objectives cannot be managed or vital robot capacities are unavailable (fatal failure).

### 3.10. ORCA

The Organic Robot Control Architecture (ORCA) aims to create a comprehensive system by combining task-specific subsystems [135]. These subsystems can be supervised by others to optimize system performance. ORCA employs two module types: Basic Control Units (BCUs) for fundamental operations like motor control and Organic Control Units (OCUs) that observe and potentially modify BCUs’ behavior. OCUs rely on a health signal to rate the fitness of the BCU [136]. ORCA supports complex tasks through grouped modules and hierarchical layers.

ORCA [137,138] integrates various hardware and software approaches to maintain system control, safety, and efficiency, even in unexpected scenarios such as faults or changing environments.

**Figure 15:**
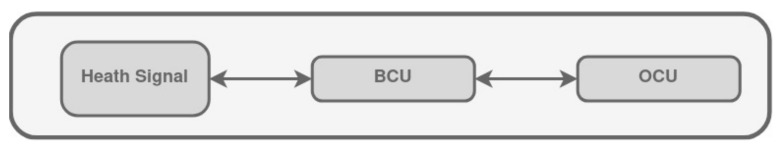
ORCA concept. Adapted from [135].

The ORCA class diagram (Figure 15) defines BCUs and OCUs as interfaces, while health signals are classes. Modules connect within the same layer through the “Neighbors” property and create hierarchies using the “Parent” and “Children” properties. Specific interfaces facilitate the connection between OCUs and BCUs, ensuring appropriate observation.

Another aspect within ORCA is the concept referred to as the “Parameterize method”. This method anticipates a BCU that shares the same type as the class in which it is implemented and produces a modified version of the given BCU. This method operates solely within a BCU when no OCU is attached. If an OCU is attached, control is passed to the OCU’s “Parameterize” method, enabling the OCU to make adjustments to its observed BCU.

The “Health Signal” class embodies the hierarchical aggregation of various signals into a collection of interconnected health signals. The “Combination Logic” property is of the delegate type “Logic Method” nested within it, permitting tailored specifications for computations that align with specific task requirements. By default, the “Combination Logic” is configured to compute the mean of all linked health signals.

### 3.11. LAURON Control Architecture

**Figure 16:**
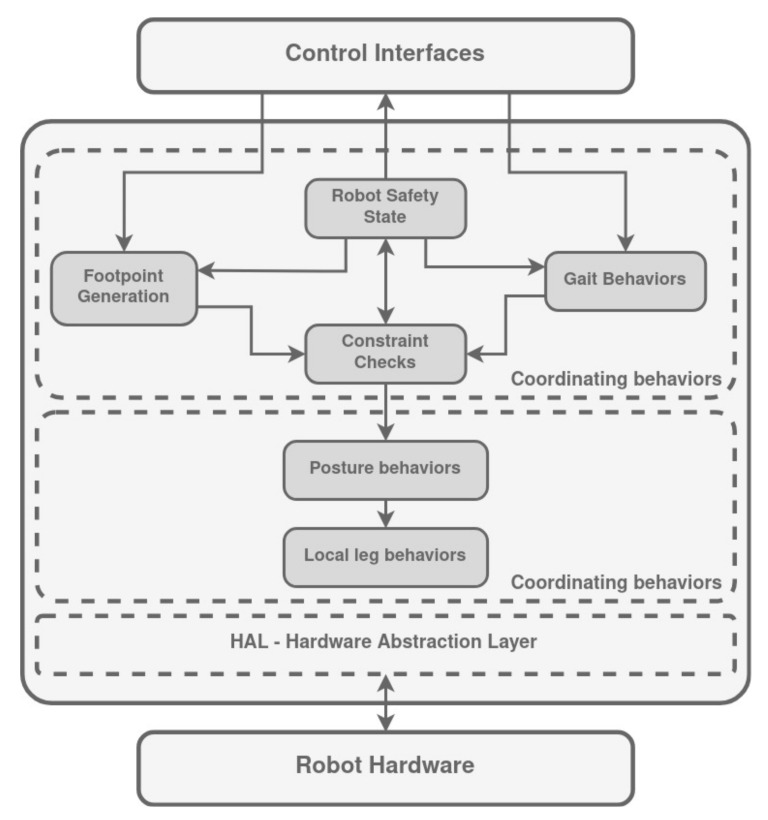
LAURON V control architecture. Adapted from [139].

LAURON V is a legged robot controlled by a behavior-based modular design approach (Figure 16). It subdivides the system into understandable hierarchical layers and small individual behaviors [8]. The layers are (a) the Hardware Architecture, (b) the Hardware Abstraction Layer (HAL), and (c) the Behavior-Based Control System.

The Hardware Architecture consists of the LAURON on-board PC, which offers sufficient computational power to run all the software, the behavior-based control system, and the hardware abstraction layer, on the robot. As well as in LAURON IV, which is controlled by a similar behavior-based control architecture [139], the low-level PID joint controllers work on custom motor controller boards (UCoM—Universal Controller Modules [140]) that are connected to the control PC via the CAN bus interface. The control center, which includes a 3D model of the robot and all relevant control interfaces, is executed on an independent external PC.

LAURON’s HAL is used to filter data, multiplex and convert values, and solve the direct and inverse kinematics. Calculating the inverse kinematics, IK, is a rather complex part in the HAL. By placing HAL between the behavior-based control system and low-level motor controllers, the control systems remain largely robot-independent [8].

In relation to the Behavior-Based Control System, external inputs specifying desired velocities and walking patterns serve as the foundation. These inputs may originate directly from the control center or stem from higher-level software components such as path planning or mission control. Foot point-generating behaviors engage in the computation of vital parameters including the Anterior Extreme Position (AEP), Posterior Extreme Position (PEP), swing height, and the ensuing step cycle. Gait behaviors take on the role of stimulating the swing or stance behavior for each leg, orchestrating leg movements to produce the intended patterns. The process of behavior fusion is entrusted with facilitating seamless transitions and intelligent amalgamation of various values. For instance, the step size (AEP and PEP) and step frequency are meticulously incremented by small deltas in each walking step, progressively converging towards the desired overall robot velocity.

The parameters intrinsic to these behaviors, together with motivational signals, serve as foundational elements in generating leg and body movements within local leg behavior groups. Each self-contained local leg behavior group comprises three distinct behaviors: swing, stance, and reflex (which handles collision detection and ground contact). The initiation of swing and stance behaviors is propelled by the walking pattern behavior, while reflexes are triggered directly by the actions of swing and stance behaviors and subsequently activated by sensory input, such as motor currents or ground contacts. Posture behaviors come into play, introducing shifts and offsets that exert control over the body’s inclination, height, and position. All of these diverse behaviors synergize their outputs, culminating in the determination of both position coordinates and orientation angles for each leg. The Inverse Kinematics (IK) process, housed within the Hardware Abstraction Layer (HAL), stands responsible for deducing the relevant joint angles. Concurrently, the Universal Controller Module (UCoMs) system governs motor control, while also acquiring data from internal sensors.

### 3.12. Nimble Limbs Architecture

**Figure 17:**
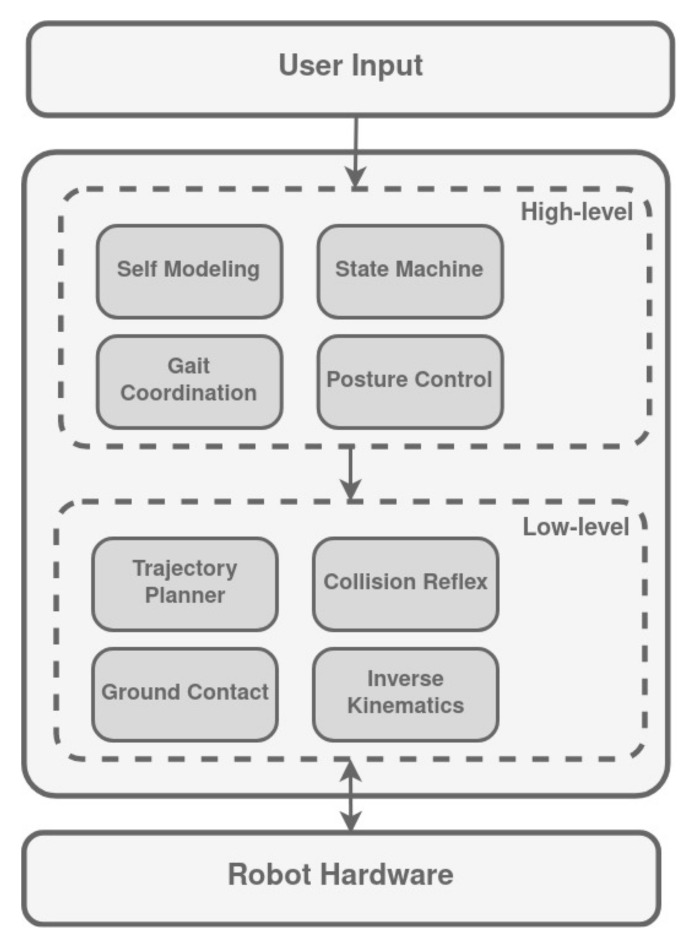
Nimble Limbs control architecture. Adapted from [40].

Nimble Limbs architecture was inspired by living beings such as ants, which are required to carry heavier objects cooperatively. It is presented as a decentralized architecture for controlling legged robots, and is divided into two layers (Figure 17), where the higher level is executed by the master unit (one of the legs), and the low level is executed on the CPU of the individuals of the legs. Thanks to the master unit, the communication need is greatly reduced since only the most necessary control values are transmitted to the lower levels. However, losing the master leg would prove fatal for any setup and greatly puts the robot and payload at risk.

The higher level contains the tasks of self-modeling, gait coordination, and posture control. In addition, it generates a state machine that controls overall behavior. On the other hand, the lower level includes the trajectory planner, the ground contact behavior, the collision checker, and the inverse kinematics controller.

### 3.13. MoCLORA

**Figure 18:**
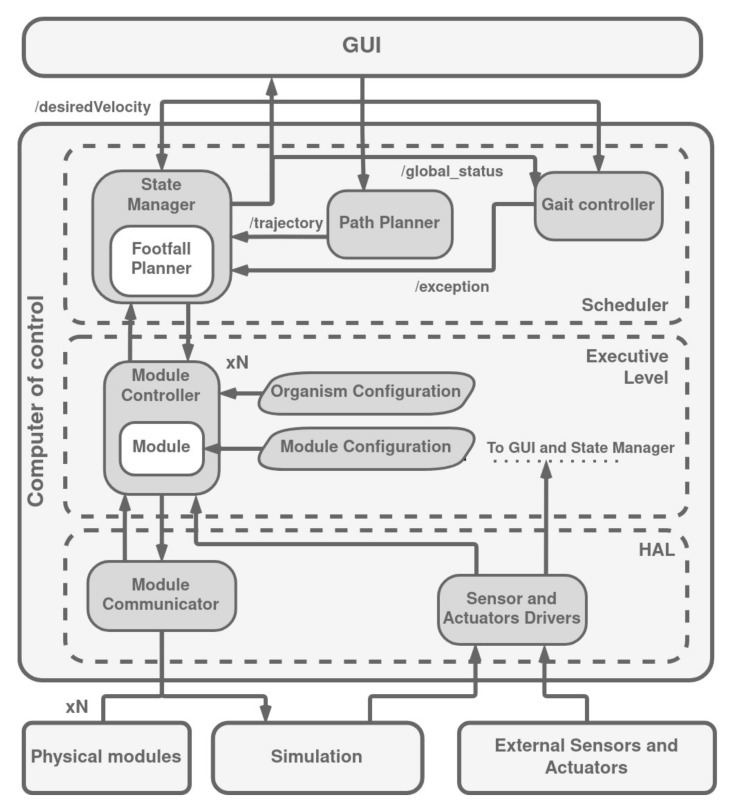
MoCLORA architecture. Adapted from [26].

The Modular Climbin and Legged Robotic Organism Architecture [26] arises to control a modular legged and climbing robotic organism. The authors improve the performance of the behavior-based architecture for the robot ROMHEX [28] and extend its use to the ROMERIN robot [11,12]. MoCLORA is implemented in C++ and uses ROS2 communication tools to share information between architecture components and devices (Figure 18). It is designed for a general robotic organism composed of leg-shaped robots with the following requirements:

- It seeks to imitate the behavior of animals, specifically those of legged insects.
- Modules support an intentional movement expressed by the body.
- The number of modules can be variable.
- The components of the robot can be replaced by virtual components (digital twin).

In the upper part of the architecture, the Graphical User Interface (GUI) is found. This component communicates only with the CC, which is the core of the control architecture and is divided into three layers: HAL, Executive Level, and Scheduler. In the lower part, external devices are found. These devices, where the robotic organism is found, can be physical or simulated.

The HAL, Hardware Abstraction Layer [141], serves to separate the control units of the modules from the modules themselves, effectively providing a mechanism to mask complexity when systems become overly intricate for efficient management. Controllers for the modules dispatch instructions to their respective systems and gather necessary feedback, all while being oblivious to whether they are interacting with actual modules or simulated ones. The responsibility of the Module Communicator is to ensure that messages are correctly directed, facilitating seamless and transparent communication. Essentially, the HAL layer ensures that upper levels can operate without needing to distinguish whether communications are directed toward the physical modules of the robotic entity or its digital counterpart’s modules.

Messages may originate from a superior level, where they define a command that a module is tasked with executing. These instructions are then forwarded to the targeted module, identified through its communication details, usually comprising the IP address and port number. Conversely, updates regarding the current state of a specific module can be transmitted back through any of the designated channels.

In this layer, different Sensor Drivers can be located to isolate the sensors’ connection and protocols from higher layers. Any sensor driver can be found, but it is recommended to have at least an IMU for gravity vector detection and a camera for environment visualization.

The Executive Level encompasses the elements that oversee module operations from an elevated standpoint. It consists of *N* Controller Nodes, which are dynamically instantiated based on the setup of the organism. Each node is equipped with a Module Controller tasked with determining the configuration of the modules in alignment with the target body position. This includes the Module object, which directly manages a module’s components—namely actuators, sensors, and suction devices. It also entails calculating gravity compensation [142], as well as handling the forward and inverse kinematics and dynamics of a module, referred to as FK, IK, FD, and ID, respectively.

Therefore, the Module Controller leverages the functionalities provided by the Module object to manage it in line with the requirements for body movement. The Module Controller consults the Organism Configuration file, which specifies the total number of modules, their identifiers, spatial orientation relative to the body’s center, IP addresses, ports, and physical characteristics of the body (such as maximum allowed speed, mass, and inertia). Conversely, the Module object derives its information from the Module Configuration, which outlines the kinematic and dynamic attributes specific to a module, limitations of its joints, the peak velocity and acceleration of the Tool Center Point (TCP), parameters for calculating inverse kinematics, and the conversion metrics for translating between normalized joint positions and the readings from the actuators’ encoders.

The uppermost layer, referred to as the Scheduler, takes on the task of crafting a trajectory in response to user commands, thereby determining the optimal sequence of body movements and defining the locomotion pattern.

In order to achieve this, the Path Planner creates a velocity profile based on the instructions received from the user. With this profile at hand, the State Manager Node issues directives to the subordinate level. Internally, the State Manager calculates the necessary body positioning to adhere to the planned trajectory and estimates the robot’s state (which can be implemented as indicated in [143]), the COM of the entire robotic organism, and the next position where a module should step. One of the main duties of the State Manager is to take into account the static and gripping stability of the commanded positions of the robot organism based on the modules and body positions, direction of gravity, and reaction forces computed in the suction cups.

Within the State Manager, the Footfall Planner selects the best position in which the leg in the swing phase has to step to ensure safety and comfort. It can optimize a function, or generate a predefined sequence of movements as a Central Pattern Generator (CPG).

The Gait Controller is responsible for generating the swing/stance phases. It constantly checks the global status of the robot to detect uncomfortable positions according to leg position, torque, and suction cup status. When this situation is detected, the Gait Controller throws an exception to stop the motion and plan a new trajectory or move one of the conflicting legs.

A Graphical User Interface (GUI) is a digital interface in which the user interacts with graphical components such as icons, buttons, and menus. In a GUI, the visuals displayed in the user interface convey information relevant to the user, as well as actions that they can take. The GUI typically controls the robotic organism at a higher level, detects malfunctions, moves the robot, operates over the environment, and visualizes the robot status and surroundings.

## 4. Comparative of the Control Architectures

Table 3 compares the control architectures described previously. It includes the applications where architectures have been tested, a short analysis, the year of the first publication, and the number of layers.

In this article, we include our own proposal to analyze control architectures through a series of features. A rating that goes from 0 to 10 is given for each control architecture:

1. Modularity. It is the degree to which a system’s components may be separated and recombined, often with the benefit of flexibility and variety in use. An architecture that is qualified with 0 is considered to be extremely difficult to recombine in a modular way, whereas an architecture that is qualified with 10 is fully modularly designed and components can be placed, recombined, and flexibly used. An architecture qualified with a middle rating would be one in which some components can be reused, separated, and combined, but modularity is not the main objective of the architecture, or it fails in its implementation.
2. Robustness. It is the ability to withstand or overcome adverse conditions or rigorous tests. An architecture qualified with 0 is considered to be weak under unexpected circumstances, whereas an architecture qualified with 10 is strong against failures and noise. For example, a legged robot can react to the loss of a joint and reconfigure itself to continue to be able to perform its intended tasks. An architecture qualified with 5 would be the one that overcomes a few of the unforeseen scenarios or attempts to cover many of them but fails in the attempt.
3. Implementability. It is the quality of being implementable and the quality or condition of being plain or uncomplicated in form or design. An architecture that is qualified with 0 is considered extremely difficult or even impossible to implement into a real system, whereas an architecture that is qualified with 10 is easy and very intuitive to implement. An architecture qualified with 5 would be the one that allows the developer to implement some of the components, but the entire architecture is thought for a specific target and cannot be reused.
4. Versatility. It is the ability to be adapted to many different functions or activities. An architecture that is qualified with 0 is considered extremely difficult to adapt to different applications and to include required features, whereas an architecture that is qualified with 10 is adaptable to all the needed functions. An architecture qualified with 5 would be the one that can be adapted to some specific tasks, but it fails to be implemented in a wide range of applications.
5. Explicitness. It is the quality of being expressed without vagueness, implication, or ambiguity. An architecture that is qualified with 0 is considered to be ambiguous, and implementation details are not explained in depth, whereas an architecture that is qualified with 10 is well-detailed and very explicit. An architecture qualified with 5 would be the one that expresses some items in depth, but it does not go into detail in many others or does not include important implementation details.

**Figure 19:**
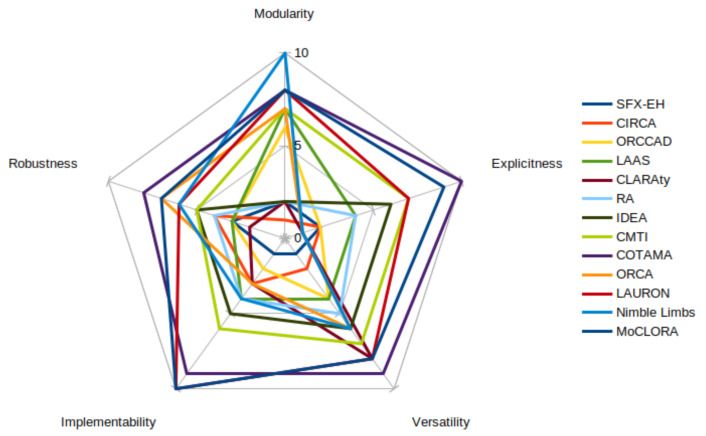
Comparative of some control architectures features (Table 4).

Figure 19 and Table 4 illustrate the analysis and rating of features within various control architectures, scaled from 0 to 10. These ratings assess the relevance and efficacy of each architecture specifically for legged, climbing, and modular robotic applications. Importantly, these evaluations are theoretical and reflect potential applicability; the architectures have not been implemented on any system, thus, no assessments are made outside this scope. This scale guides the reader in understanding the comparative advantages of each architecture, emphasizing their suitability for enhancing robotic performance in theoretical applications without the necessity of prior empirical implementation.

As illustrated in Figure 19, the implementability of the LAURON architecture, the modularity of nimble limbs, and the overall features of CMTI and, in particular, COTAMA and MoCLORA are highlighted.

The state-of-the-art control architectures for L&C robots reveal that much work remains because, although plenty of them are structurally defined, they lack a defined architecture. Although there is a lack of control architectures for L&C robots, it is possible to find those for legged robots, such as [158] or [159], which describe the motion controller of two-legged robots. Robots with more than two legs, such as [160], have a control system that only covers leg management and excludes high-level control. A similar approach is Free Gait [161], which is designed to control whole-body motions for quadrupeds and is applied to ANYmal and StarlETH robots. Other examples are the control architectures specified for the robots Ambler [15] and MECANT I [22]. These architectures are specialized and tailored to their specific operational needs, not providing a generalized framework suitable for robot control. In addition, these architectures focus on specific device integration and functional system layouts rather than an agent-based structure.

## 5. Conclusions

In this paper, we first include a review and classification of modular legged and climbing robots. Exploring one feature by one, a designation has been included for some of the robots that collect one of these characteristics. A set of tree diagrams have been designed for a better understanding of the classification.

We also present a comprehensive review of the most important control architecture in robotics for our case study, focusing on the control of modular legged and climbing robots. We chronologically included those architectures that can be used in the control of this type of robot, explaining their features, and analyzing them from a point of view of modularity, robustness, implementability, versatility, and explicitness.

This paper endeavors to not merely catalog control architectures, but to evaluate them through the lens of essential characteristics that define their efficacy in real-world applications. Through this classification, we aim to underscore the significance of modularity for adaptive and scalable systems, robustness to ensure consistent performance under uncertain conditions, implementability for practical deployment, versatility to handle diverse operational scenarios, and explicitness to facilitate understanding and troubleshooting.

## Figures and Tables

**Table 1:** Typical leg structures of legged robots. Adapted from [7].

Type	Sketch	Advantages	Disadvantages	Examples
Articulated leg	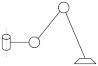	High maneuverability and flexibility	Reverse articulated torque under the unreasonable walking way	LAURON V [8], ANYmal [9], ATHLETE [10], ROMERIN [11,12]
	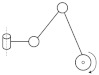	High maneuverability, high mobile speed, and high energy efficiency	Reverse articulated torque under the unreasonable walking way, difficult to control, and low versatility	Momaro [13], PAW [14]
Orthogonal leg	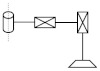	Gravity decoupling	Low flexibility	Ambler [15], ROBOCLIMBER [16], H. Montes hexapod [17]
Pantograph leg	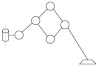	The horizontal and vertical motions are decoupled	High peak power requirements	SCALER [18], Oncilla [19], LARM [20], PV-II [21], MECANT I [22]
Telescopic leg	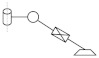	Compact structure	Low energy efficiency	Mainly for biped robots [23,24]

**Table 2:** Modular robots classification. Gray color represents represents the existence of the given feature.

Robot	Mechanically Homogeneous	Intramodule Communication	Power Sharing	Decentralized Control	Self-Configuring
ine Polybot					
ine Crystalline					
ine Conro					
ine M-TRAN					
ine Telecube					
ine ATRON					
ine Microtub					
ine Superbot					
ine Molecubes					
ine Odin					
ine Roombot					
ine iMOBOT					
ine Ubot					
ine Transmote					
ine M3 Express					
ine CoSMO					
ine Kairo 3					
ine Hinged-Tetro					
ine Fable II					
ine TR:R					
ine Ani-Bot					
ine Snapbot					
ine SB blocks					
ine NL					
ine Morphius					
ine WalkingBot					
ine MLS					
ine KARAKASA					
ine ROMERIN					

**Table 3:** Comparative of the control architectures.

Name	Applications	Analysis	Year	Number of Layers
SFX-EH	Sensor fusion and failure detection on robots, such as Clementine 2 [144]	It requires only a partial causal model of sensing failure, and the control scheme strives for a fast response. However, it is (a) an old architecture with obsolete approaches, (b) only applicable to sensor failure treatments, and (c) difficult to implement on a high scale and in complex systems with the objective of robustness and fault tolerance. It has a lack of fault handling in many situations and high limitations in new control systems.	1992	2
CIRCA	Heathkit Hero 2000 [145]	It is an approach focused mainly on control-level goals, not on task-level goals. It is thought to produce a precise, high-confidence response in a timely fashion to a limited set of inputs. Thus, the environment and the problem should be well-known and defined.	1993	3
ORCCAD	Autonomous vehicles [146]	There are two different types of modules, RT performs the task and RP defines the interface of RT. It contemplates error types and simple fault tolerance techniques. However, as well as ORCA, the complexity of the system may increase exponentially with new fault tolerance techniques, while the organization structure may become untenable.	1996	2 ^1^
LAAS	DALA [147], iRobot ATRV [148], HRP2 [149], Rackham [150], Jido [151]	Similarly to CMTI, the division in three layers, where the middle one ensures the system safety, improves the robustness. However, it is very open, where it is the user who has to apply more fault tolerance techniques.	1998	3
CLARAty	PDM [152,153], Rocky 7 [154] and 8 [155]	The suppression of a layer increases the granularity as a third dimension. However, the tasks of the executive and planner are fuzzy. It gives more freedom to the researcher but less control architecture definition.	2001	2
RA	Deep Space 1 [156]	Thought for planned tasks, where the main planner is a state machine with predefined actions.	2002	3
IDEA	Deep Space 1, spacecrafts [156]	Improvement of RA through the use of tokens. It combines reactive and deliberative control, depending on the problem scope, giving more robustness than RA.	2002	3
CMTI	AUV TAIPAN [132]	Good architecture for improving with fault tolerance mechanisms. The structure is simple, but it covers a high amount of situations.	2006	3
COTAMA	Mobile robots [157]	Complex architecture that covers a huge quantity of situations and problems, becoming the most fault-tolerant architecture included in this article. It includes the capabilities of ORCA (with the addition of observer modules) and CMTI, but it improves reliability and robustness respecting the others.	2010	2
ORCA	OSCAR [36]	The idea of dualizing the modules covers a large amount of problems, however, the complexity of the system may increase exponentially with fault tolerance techniques implementation (which are not contemplated in the architecture), while the organization structure may become untenable.	2012	Non defined
LAURON	LAURON IV [139], LAURON V [8]	It is a good approach thought for legged robots with a variable number of legs. It contemplates all the features of the walking process, however, it does not do so for external perturbations.	2014	3
Nimble Limbs	Nimble Limbs system [40]	It proposes a decentralized control with a variable number of legs, but it is still preliminary and it has not been tested in a physical system to validate its behavior. It is simple, and in this way, it does not contemplate many scenarios, or communication details and also does not go into implementation details.	2019	2
MoCLORA	ROMERIN [26]	It proposed an approach to control modular legged robots, including a torque-based control for the position and velocity control for the robot body and legs. It includes leg coordination, state estimation, gait controller, path and footfall planning, and collision avoidance.	2023	3

^1^ (Authors present three layers, but the application layer can be considered out of the control architecture).

**Table 4:** Comparative of the control architectures.

Name	Modularity	Robustness	Implementability	Versatility	Explicitness
SFX-EH	2	3	1	1	2
CIRCA	1	4	3	2	2
ORCCAD	6	3	2	4	2
LAAS	7	3	4	4	4
CLARAty	2	2	3	8	1
RA	2	4	4	5	4
IDEA	2	5	5	6	6
CMTI	7	5	6	7	7
COTAMA	8	8	9	9	10
ORCA	7	7	3	6	1
LAURON	8	6	10	8	7
Nimble Limbs	10	6	4	6	1
MoCLORA	8	7	10	8	9
